# Burden of migraine and unmet needs from the patients’ perspective: a survey across 11 specialized headache clinics in Korea

**DOI:** 10.1186/s10194-021-01250-6

**Published:** 2021-05-24

**Authors:** Byung-Kun Kim, Min Kyung Chu, Soo Jin Yu, Grazia Dell’Agnello, Jeong Hee Han, Soo-Jin Cho

**Affiliations:** 1 Department of Neurology, Nowon Eulji Medical Center, Eulji University School of Medicine, Seoul, South Korea; 2 Department of Neurology, Severance Hospital, Yonsei University School of Medicine, Seoul, South Korea; 3 Lilly Korea Ltd., Seoul, South Korea; 4 Eli Lilly and Company, Indianapolis, IN USA; 5 Department of Neurology, Dongtan Sacred Heart Hospital, Hallym University College of Medicine, Hwaseong, South Korea

**Keywords:** Migraine, Korea, Burden, Disability, MIDAS, MSQv2.1, Diagnosis, Treatment, Patient survey

## Abstract

**Background:**

Migraine is a neurological, primary headache disorder affecting more than 1 billion people worldwide, with a multi-faceted burden that can significantly impact the everyday life of a patient, both during and between attacks. However, studies on patient awareness, burden, and clinical management of migraine in Korea are limited and outdated. The aim of this study was to comprehensively investigate the current difficulties and unmet needs that Korean patients with migraine encounter from their perspective.

**Methods:**

A total of 207 patients with episodic or chronic migraine aged between 15 and 76 years, completed a survey designed to cover the following topics: diagnosis, understanding of the disease, treatment experience, disability, and quality of life. Patients were recruited by their neurologists from 11 specialized headache clinics in Korea and completed the survey between 22 July and 19 August 2019. Validated scales such as the Migraine Disability Assessment (MIDAS) questionnaire and Migraine-Specific Quality of Life Questionnaire version 2.1 (MSQv2.1) were used to assess levels of disability and quality of life, respectively, in patients.

**Results:**

On average, it took 10.1 years from onset of symptoms to diagnosis and a mean of 3.9 hospitals were visited for treatment prior to the patient’s current hospital. There was a lack of understanding among respondents about migraine, with 55.6% believing that unilateral headache is a unique feature of migraine compared with other headache disorders. On average, high levels of disability and poor quality of life were reported by patients, as assessed by MIDAS and MSQv2.1, respectively, but only 23.7% had regularly taken preventive medication in the past. Overall satisfaction with previous doctor-patient relationships was reported by 29.5% of respondents, and satisfaction with preventive and acute medications by only 40.8% and 27.1% of the respondents, respectively.

**Conclusion:**

Korean patients with migraine experience significant disability and reduced quality of life as a result of the disease and have clear unmet needs in terms of diagnosis, understanding of the disease, and disease management including treatment.

**Supplementary Information:**

The online version contains supplementary material available at 10.1186/s10194-021-01250-6.

## Background

Migraine is a disabling, neurological disease that can severely impact every aspect of an individual’s life, yet is still under-recognized, under-diagnosed, and under-treated [1]. Migraine affects 11.6% of the world’s population [2], or approximately 1.3 billion people [3] and is the leading cause of disability in persons under 50 years of age [4]. Migraine and the accompanying symptoms can be significantly burdensome to patients, impacting daily functioning ability and quality of life both during and between migraine attacks [5–9]. Non-headache symptoms and co-morbid disorders such as depression and anxiety are common with migraine and can further hinder management of the disease [7, 10–14]. Migraine has a two- to three-fold higher prevalence in women compared with men [2, 15]. The median prevalence of migraine in the Asia-Pacific region is 9.1% [16]. In South Korea, the estimated prevalence is approximately 8 to 9% in women and 3% in men [11, 17, 18], and 6% overall [16, 18, 19]. The age group with the highest prevalence is 40–49 followed by 30–39 years of age [18, 19]. A recent study on sex differences in migraine prevalence among Korean patients showed that in women, prevalence is highest in the 30–39 age group followed by 40–49 [11].

Studies in East Asia, including South Korea, have demonstrated that migraine is associated with a significant burden for patients. Migraine attacks and accompanying symptoms such as nausea, vomiting, and sensory disturbances impact significantly on daily activities (work, school, chores) and overall quality of life [18–25]. In addition, there are unmet needs for East Asian patients in relation to diagnosis and treatment, owing in part to lack of the following: sufficient and appropriate diagnosis, disease awareness, and use of prescription medication [22, 23, 25, 26]. Lack of diagnosis and disease awareness could in turn contribute to an underestimated prevalence. A survey was conducted across eight Asian countries, including South Korea, in which 222 neurologists and 3177 patients participated. The results were published in 2008 and revealed that 36% of patients had received emergency treatment for migraine. Eighty-four percent of patients were taking acute medication with 40% of those patients dissatisfied with the relief obtained within 2 h of taking the medication. Physicians reported that 71% of their patients with migraine were not taking preventive treatment, and recommended that 68% of those patients were in need of such treatment [23].

The available literature on patient awareness, burden, and clinical management of migraine in Korea is limited and outdated. Such information is crucial in ensuring a patient-centric approach in the appropriate diagnosis and treatment of affected individuals. A survey was designed to comprehensively investigate the more current difficulties and unmet needs that Korean patients face regarding migraine diagnosis, awareness, treatment, and their perceived disability and quality of life.

## Materials and methods

### Patient selection

First-visit patients diagnosed with episodic or chronic migraine according to International Classification of Headache Disorders (ICHD) criteria, and with previous treatment experience, were recruited from the following 11 specialized headache clinics in Korea: Nowon Eulji Medical Center, Gangbuk Samsung Hospital, Korea University Guro Hospital, Dongtan Sacred Heart Hospital, Bundang Jesaeng Hospital, Samsung Medical Center, Seoul Paik Hospital, Seoul Medical Center, Severance Hospital, Uijeongbu St. Mary’s Hospital, and Ilsan Paik Hospital. Conducting of surveys is subject to Institutional Review Board exemption in Korea, but approval is required for preparation of a publication. Prior to participation, patients provided written consent to use the results of the survey for statistical purposes. Approval for preparation of this manuscript was granted by the Institutional Review Board of Nowon Eulji Medical Center (Approval No 2020–06-009). Survey data were handled confidentially, and anonymity of respondents was maintained throughout the study.

### Survey design and outcomes

The survey was created in collaboration with Hankook Research Ltd. and included questions on the following in relation to migraine specifically: history and diagnosis, knowledge about migraine, utilization of medical services, disability and quality of life, unmet treatment needs regarding disease management, and experience with preventive and acute medications prior to visiting their current hospital. Validated scales such as the Migraine Disability Assessment (MIDAS) questionnaire [27] and the Migraine-Specific Quality of Life Questionnaire version 2.1 (MSQv2.1) [28] were used to assess the level of disability and quality of life of patients, respectively. The MIDAS was developed to assess headache-related disability with the aim of improving migraine care [27]. It is a self-administered questionnaire designed to quantify headache-related disability over a 3-month period. This questionnaire consists of five questions pertaining to time or productivity lost, as well as the limited ability to participate in work or school, household chores, family events, and social or leisure activities. The question responses are in the form of number of days affected in the past 3 months. The total MIDAS score, which is the summation of the answers for each question, corresponds to a grade of migraine-related disability (0–5, Grade I, minimal or infrequent disability; 6–10, Grade II, mild or infrequent disability; 11–20, Grade III, moderate disability; ≥21, Grade IV, severe disability). The MSQv2.1 measures quality of life among migraine patients during the previous 4 weeks [28]. It has three scales assessing unique quality of life domains: Role Function-Restrictive (RR), Role Function-Preventive (RP), and Emotional Function (EF) which consist of different items to assess. These items include; limitations of patients’ performance of normal activities (for RR), interruptions of patients’ performance of normal activities (for RP) and impact of migraine on the respondents’ emotions, such as frustration or helplessness (for EF). The item responses range from 1 to 6 (1 = none of the time; 2 = a little bit of time; 3 = some of the time; 4 = a good bit of the time; 5 = most of the time; 6 = all of the time). All items are reverse-coded and standardized to a 0 to 100 scale. Thus, higher scale scores indicate better migraine-related quality of life [29].

Data were described descriptively. Categorical variables were reported using percentage. Numerical variables were reported using the mean and either standard deviation (SD) or standard error (SE), calculated using Microsoft Excel for Office 365. No formal statistical analyses were conducted.

### Completion of survey

The total number of patients who completed the survey was 207. The survey was completed by the patient in an approximately 20 min period, in the presence of an educated guide. Surveys were completed between 22 July and 19 August 2019.

## Results

### Respondent demographics

Respondent demographics are shown in Table 1. Out of the 207 respondents, the average age was 45.5 years old and the category with the largest number of patients was 40–49 years of age, followed by 50–59, 30–39, 60+, and 10–29. Overall, the vast majority were female, mean age of onset of migraine was 27.7 years, and mean time from first symptoms to diagnosis was 10.1 years. Mean age of onset and mean time from first symptoms to diagnosis varied significantly between age groups. The overall mean duration of disease was 17.7 years. The overall mean number of headache days per month was 12.4 and the majority of patients had episodic migraine (Table 1).

**Table 1:** Respondent demographics

	10 - 29^**a**^	30–39	40–49	50–59	60 + ^**b**^	Overall – all age groups combined
***Variable***						
*Number of patients, N (% of total patients)*	25 (12.1%)	40 (19.3%)	63 (30.4%)	47 (22.7%)	32 (15.5%)	207 (100%)
*Female, n (% of age group)*	15 (60.0%)	33 (82.5%)	55 (87.3%)	45 (95.7%)	27 (84.4%)	175 (84.5%)
*Mean age, years (SD)*	24.3 (4.1)	34.7 (3.2)	44.7 (2.9)	54.3 (2.7)	64.0 (3.5)	45.5 (12.6)
*Mean age of onset, years (SD)*	17.0 (3.5)	20.4 (7.4)	27.5 (11.1)	30.8 (11.9)	41.1 (14.5)	27.7 (12.9)
*Mean age of diagnosis, years (SD)*	21.4 (4.3)	30.9 (5.3)	36.6 (7.7)	43.4 (10.7)	53.5 (12.1)	37.8 (12.9)
*Mean time from first symptoms to diagnosis, years*^c^	4.4	10.5	9.1	12.6	12.4	10.1
*Mean duration of disease, years (SD)*	7.3 (4.1)	14.2 (7.6)	17.2 (11.5)	23.6 (11.7)	22.9 (14.1)	17.7 (11.8)
*Mean number of headache days per month (SD)*	11.6 (9.6)	11.6 (9.6)	10.8 (7.8)	14.0 (9.6)	14.8 (11.8)	12.4 (9.5)
*Episodic migraine, n (% of age group)*	17 (68.0%)	27 (67.5%)	45 (71.4%)	28 (59.6%)	18 (56.3%)	135 (65.2%)
*Chronic migraine, n (% of age group)*	8 (32.0%)	13 (32.5%)	18 (28.6%)	19 (40.4%)	14 (43.8%)	72 (34.8%)

Demographics are presented for each age group and for overall respondents, the latter inclusive of all age groups.

^a^Three respondents were under 18 years of age; 2 respondents were 15 years of age and 1 respondent was 16 years of age. All other respondents in this group were 22–29 years of age.

^b^Two respondents were aged 70 and 1 respondent was aged 76. All other respondents in this group were 60–68 years of age.

^c^Values for each group = mean age of diagnosis – mean age of onset. Hence, SD were not calculable for this variable.

*N / n* Number of patients, *SD* Standard Deviation

### Knowledge about migraine

**Fig. 1:**
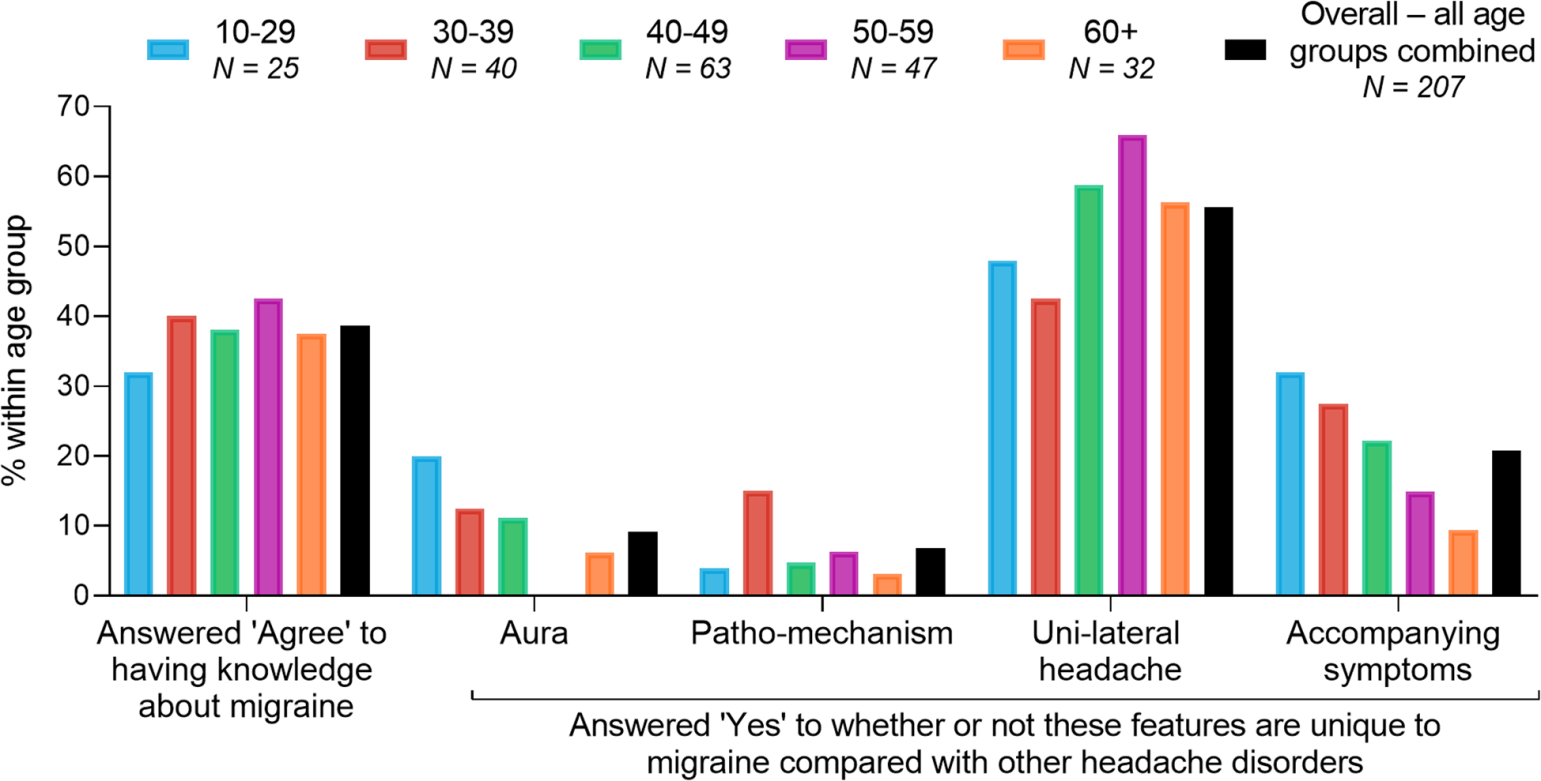
Knowledge about migraine. Results for knowledge of migraine are presented for each age group and for overall respondents, the latter inclusive of all age groups. Results are presented as percentage of respondents. N = Number of patients

With regards to knowledge about migraine, less than half of patients overall believed that they had some level of knowledge (Fig. 1). When questioned about differences between migraine and other headache disorders, few patients overall believed that migraine was different from other headache disorders in terms of aura, patho-mechanism, and accompanying symptoms. Fewer patients in the older age groups compared with younger age groups believed that aura and accompanying symptoms were unique features of migraine. Overall, approximately half of patients regarded unilateral headache as a unique feature of migraine (Fig. 1).

### Utilization of medical services for migraine and cost of medication

Results related to utilization of medical services and cost of medication are presented in Table 2. Among respondents, a mean of approximately four hospitals were visited for migraine treatment prior to the current hospital. There was a greater mean number of visited hospitals in older compared with younger patients. Representation of these data by MIDAS grade rather than age group revealed that those in MIDAS grades III and IV had reported the highest mean number of hospitals visited (4.4 [SE=1.3] and 4.1 [SE=0.4], respectively), compared with those in grades I and II (2.1 [SE=0.5] and 3.3 [SE=1.6], respectively). Approximately half of respondents overall regularly visited hospital in the past for treatment of migraine. Overall, patients spent on average 1,432,500 Korean Won a year on medication for migraine, with those over 60 spending notably less than other age groups. The majority of patients had a history of visiting a neurology clinic. Approximately half of the respondents had experience of visiting an emergency room (ER), and a quarter had experience of hospitalization. Among Magnetic Resonance Imaging (MRI), Computerized Tomography (CT), Transcranial Doppler (TCD), and Electroencephalogram (EEG), MRI was the most common diagnostic test for migraine, followed by CT, TCD and EEG. There was a notable increase with MRI as a diagnostic test with increasing age group. Approximately one-third of patients overall were satisfied with the doctor-patient relationship in previous hospitals, with notably less satisfaction in the 50–59 group compared with other age groups. Specifically, satisfaction was highest for explanation of migraine offered and lowest for effectiveness of treatment.

**Table 2:** Prior utilization of medical services and cost of medication for migraine

	10–29	30–39	40–49	50–59	60 +	Overall – all age groups combined
***Variable***						
*Number of patients, N (% of total patients)*	25 (12.1%)	40 (19.3%)	63 (30.4%)	47 (22.7%)	32 (15.5%)	207 (100%)
*Mean number of hospitals visited excluding current one (SE)*	2.6 (0.4)	3.1 (0.6)	4.1 (0.9)	4.6 (0.8)	4.6 (1.2)	3.9 (0.4)
*Regular past visits to hospital*^*a*^*, n (% of age group)*	11 (44.0)	21 (52.5)	26 (41.3)	24 (51.1)	15 (46.9)	97 (46.9)
*Mean annual cost of medication in Korean Won, million (SE)*	1.4 (0.5)	1.5 (0.3)	1.5 (0.2)	1.7 (0.3)	1.0 (0.2)	1.4 (0.1)
*Experience of:*						
*Visiting a neurology clinic, n (% of age group)*	17 (68.0)	27 (67.5)	43 (68.3)	36 (76.6)	24 (75.0)	147 (71.0)
*ER visit, n (% of age group)*	16 (64.0)	21 (52.5)	24 (38.1)	26 (55.3)	13 (40.6)	100 (48.3)
*Hospitalisation, n (% of age group)*	9 (36.0)	8 (20.0)	14 (22.2)	11 (23.4)	8 (25.0)	50 (24.2)
*Diagnostic tests*						
*MRI, n (% of age group)*	11 (44.0)	22 (55.0)	41 (65.1)	33 (70.2)	23 (71.9)	130 (62.8)
*CT, n (% of age group)*	12 (48.0)	23 (57.5)	32 (50.8)	26 (55.3)	14 (43.8)	107 (51.7)
*TCD, n (% of age group)*	8 (32.0)	18 (45.0)	26 (41.3)	23 (48.9)	11 (34.4)	86 (41.6)
*EEG as a diagnostic test, n (% of age group)*	10 (40.0)	13 (32.5)	21 (33.3)	21 (44.7)	12 (37.5)	77 (37.2)
*Satisfaction with prior doctor-patient relationships, n (% of age group)*						
*Overall*	8 (32.0)	12 (30.0)	20 (31.8)	10 (21.3)	11 (34.4)	61 (29.5)
*Explanation of migraine*	10 (40.0)	13 (32.5)	27 (42.9)	13 (27.7)	11 (34.4)	74 (35.8)
*Emotional support*	11 (44.0)	11 (27.5)	22 (34.9)	12 (25.5)	14 (43.8)	70 (33.8)
*Effective treatment*	5 (20.0)	11 (27.5)	17 (27.0)	12 (25.5)	11 (34.4)	56 (27.1)
*Devotion of time*	9 (36.0)	11 (27.5)	19 (30.2)	13 (27.7)	14 (43.8)	66 (31.9)

Results for number of prior hospitals visited excluding the current one, regular past visits to hospital, annual cost of medication, medical services used, diagnostic tests used, and satisfaction with prior doctor-patient relationships are presented for each age group and for overall respondents, the latter inclusive of all age groups

^a^Respondents who did not visit hospital regularly only visited when they were experiencing a headache. Those who visited regularly did so even in the absence of a headache and on average once every two months

*ER* Emergency Room, *MRI* Magnetic Resonance Imaging, *CT* Computerized Tomography, *TCD* Transcranial Doppler, *EEG* Electroencephalogram, *N / n* Number of patients, *SE* Standard Error

### Migraine-related disability, quality of life, and pain severity

**Fig. 2:**
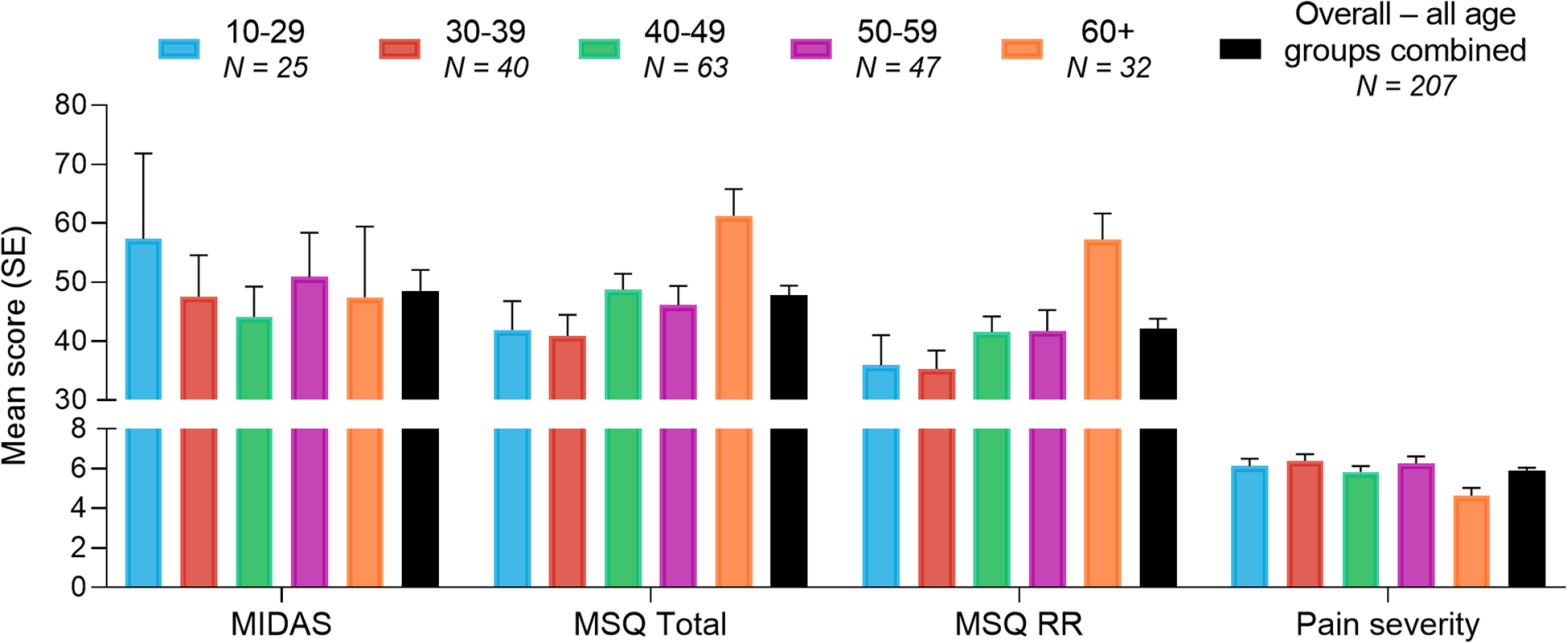
Migraine-related disability, quality of life, and pain severity. Results for MIDAS, MSQ total, MSQ RR, and pain severity are presented for each age group and for overall respondents, the latter inclusive of all age groups. Results are presented as mean score (SE). MIDAS = Migraine Disability Assessment; MSQ = Migraine-Specific Quality of Life Questionnaire; RR = Role Function-Restrictive; N = Number of patients; SE = Standard Error

Disability and quality of life were assessed with MIDAS and MSQv2.1, respectively. As revealed by MIDAS, the overall mean number of headache days in the previous 3 months reported by respondents was 37.2 (SE = 2.0). The overall mean MIDAS score was 48.4 (Fig. 2). The overall mean MSQ total score was 47.7, MSQ RR was 42.1, MSQ RP was 54.2, and MSQ EF was 52.2. Using a 0–10 numeric rating scale, with 10 representing the most severe level of pain, overall mean pain severity was reported at 5.9 (Fig. 2). Some aspects of quality of life, specifically the MSQ total and RR domain scores, were notably higher and the pain severity notably lower in the 60+ group compared with all other age groups (Fig. 2). Narrative descriptions of migraine pain, psychological difficulties, emotional effects, difficulties arising from accompanied symptoms, and daily life provided by respondents portray their personal experiences with migraine (Additional file 1).

### Past experience with preventive medication for migraine

**Fig. 3:**
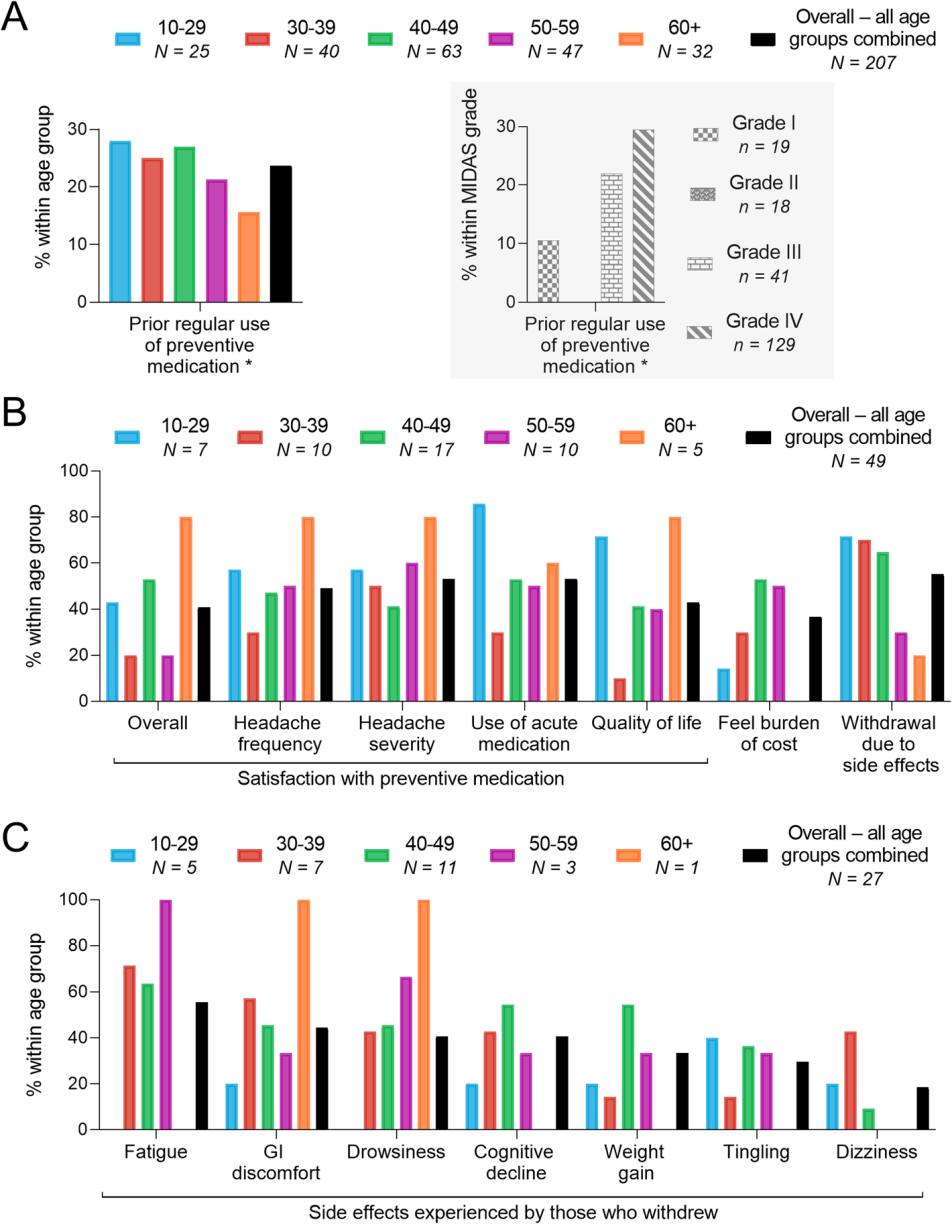
Past experience with preventive medication for migraine. Results for (**a**) prior regular use of preventive medication, (**b**) satisfaction with preventive medication, burden of cost, and withdrawal due to side effects, and (**c**) side effects experienced by those who withdrew are presented for each age group (and MIDAS grade group in (A)) and for overall respondents, the latter inclusive of all age groups. Results are presented as mean percentage of respondents. MIDAS = Migraine Disability Assessment; GI = Gastro-intestinal; N = Number of patients. * Respondents who did not take preventive medication regularly only took it when they were experiencing a headache. Those who took preventive medication regularly did so even in the absence of a headache

Approximately one-quarter of patients overall reported having used preventive medication regularly prior to visiting their current hospital (Fig. 3a)**.** Reported use or non-use of preventive medication was based on the patient’s perception and not medical and/or prescription records. The 60+ group reported the lowest levels and the 10–29 group the highest levels of prior regular use of preventive medication (Fig. 3a). Representation of these data by MIDAS grade rather than age group revealed that approximately one-third of those in the MIDAS grade IV had regularly taken preventive medication in the past (Fig. 3a; grey box). Less than half of patients with prior experience of preventive medication were satisfied overall with such treatment, with low numbers observed regarding headache frequency, headache severity, use of acute medication, and quality of life specifically. Satisfaction was also particularly low regarding burden of cost with the use of preventive medication, with a notably lower burden reported by the 60+ group compared with all other age groups (Fig. 3b). Of the 49 patients who had taken preventive medication in the past, approximately half withdrew at some point due to the side effects (Fig. 3b). Of those who withdrew, the most common side effect among those listed was fatigue, followed by gastro-intestinal (GI) discomfort, drowsiness, cognitive decline, weight gain, tingling, and dizziness (Fig. 3c).

### Past experience with acute medication for migraine

Upon assessment of overall satisfaction with acute medication taken prior to visiting current hospitals, approximately one-quarter of respondents were satisfied overall with such treatment (Table 3). Satisfaction was higher in the 60+ group compared with all other age groups. Satisfaction was low regarding being free of both headache and accompanying symptoms within 2 h, as well as a 24-h sustained effect specifically (Table 3). Satisfaction was also particularly low regarding burden of cost with the use of acute medication, with a notably lower burden reported by the 60+ group compared with all other age groups (Table 3).

**Table 3:** Past experience with acute medication for migraine

	10–29	30–39	40–49	50–59	60 +	Overall – all age groups combined
***Variable***						
*Number of patients, N (% of total patients)*	25 (12.1%)	40 (19.3%)	63 (30.4%)	47 (22.7%)	32 (15.5%)	207 (100%)
*Satisfaction with acute medication*^*a*^*, n (% of age group)*						
*Overall*	3 (12.0)	12 (30.0)	17 (27.0)	11 (23.4)	13 (40.6)	56 (27.1)
*Free of headache within 2 h*	6 (24.0)	21 (52.5)	21 (33.3)	23 (48.9)	17 (53.1)	88 (42.5)
*Free of accompanying symptoms within 2 h*	6 (24.0)	18 (45.0)	22 (34.9)	24 (51.1)	17 (53.1)	87 (42.0)
*24-h sustained effect*	6 (24.0)	14 (35.0)	19 (30.2)	17 (36.2)	13 (40.6)	69 (33.3)
*Feel burden of cost*	6 (24.0)	10 (25.0)	17 (27.0)	12 (25.5)	4 (12.5)	49 (23.7)

Results for satisfaction with acute medication and burden of cost are presented for each age group and for overall respondents, the latter inclusive of all age groups.

^a^Acute medications included prescription-only and over-the-counter medications.

*N / n* Number of patients

## Discussion

The results of this recent survey demonstrate that there are significant issues and unmet needs for Korean patients with migraine regarding diagnosis, awareness, and treatment. These include a substantial diagnostic lag, dissatisfaction with clinical management of the disease, frequent visits to hospital, and burden of cost. The burden of disease experienced by patients is evident by their significant levels of disability, pain severity, and reduced quality of life.

The respondent population were patients in specialized headache clinics, which could not be fully representative of those with migraine in the general population. The mean age of onset in the overall population of respondents was close to 30 years of age, ranging from 8 to 61 years old. Differences between groups in the age of onset is most likely due to the difficulty of recalling memories from more than 20 years ago, and selection bias. Overall, the average time from first symptoms to diagnosis was 10.1 years. Delay in diagnosis could be due to insufficient awareness of the characteristics of migraine both by non-specialist physicians and by patients [30]. The diagnostic criteria of migraine in the ICHD appear straightforward and clear, however, due to the diversity of migraine symptoms among patients and among attacks for an individual patient, some physicians are unsure of the diagnosis of migraine. Many patients also take painkillers during the early phase of migraine attacks and, as a result, their headaches frequently do not fit the diagnostic criteria of migraine. Another possible contributory factor to delayed diagnosis is unreliable information from patients regarding their headache history. Without detailed information on the characteristics of migraine headaches and any associated symptoms experienced, accurate diagnosis can be a challenge. Lack of accurate and complete information from patients on the history of their migraine could be caused in part by limited knowledge about the disease. More than half of overall respondents believed that unilateral headache is a unique feature of migraine. This commonly shared belief among patients and possibly physicians too could be in part influenced by the fact that the Chinese character for migraine, used throughout East Asian countries, directly translates to ‘one-sided head pain’. In addition, the results of the survey show that the majority of respondents did not know that migraine differs from other headache disorders with regards to aura, patho-mechanism, and accompanying symptoms. This could have impacted diagnosis as aura and accompanying symptoms such as nausea or vomiting are used in the diagnosis of migraine according to ICHD-3 criteria. In support of this, the survey results show that the 10–29 age group was amongst the most informed about unilateral headache, aura, and accompanying symptoms, and had the shortest diagnostic lag among all age groups. It could also be the case that patients did not have knowledge about aura due to lack of experience of it. Indeed, a study has shown that only 13% of Korean patients with migraine have aura symptoms [31].

A previous study across 8 Asian countries reported that 36% of patients had visited emergency rooms due to migraine [23]. The current study reveals that, for Korean patients specifically, the proportion is much higher at 48%. MRI was the most common diagnostic test among respondents, followed by CT, possibly due to MRI being more sensitive than CT in identifying intracranial pathology, and more generally preferred for the evaluation of headaches [32–34]. Neuroimaging is not usually warranted for patients who meet the diagnostic criteria for migraine and have normal findings on a neurologic examination. Neuroimaging may also contribute to a delay in diagnosis and treatment if exact diagnosis and proper education about migraine are not subsequently provided [35]. Some patients, however, might insist on the use of neuroimaging, as it can relieve anxiety about underlying pathology and thus improve quality of life.

Assessment of the doctor-patient relationship in patients’ previous hospitals revealed that there was significant dissatisfaction among patients. Specifically, less than half of patients were satisfied with the explanation of migraine, emotional support, effectiveness of treatment, and devotion of physician’s time. In the Korean medical system, there is no method of compensation for counselling, education, and evaluation of headache severity. Along with the fact that Korean physicians usually spend no more than 10 min caring for one patient, including first-visit patients, this could in part explain the dissatisfaction with prior doctor-patient relationships. The lack of patient knowledge about migraine also revealed in the survey results could be partly related to the low satisfaction with the explanation of migraine. Studies have shown that effective communication between physicians and patients plays a role in determining diagnosis, treatment compliance and medical outcomes [36–38]. Patient dissatisfaction with this relationship is an area of concern for physicians, leading to the creation of strategies and tools to facilitate this communication network [37].

The components of the survey related to patients’ past experience with medication clearly demonstrate unmet treatment needs for Korean patients. Only 23.7% of respondents overall had regularly taken preventive medication in the past, despite reporting a mean number of 12.4 headache days per month. Such poor treatment optimization could be potentially responsible, at least in part, for the high levels of disability (MIDAS) and pain severity, along with poor quality of life (MSQ) reported by respondents. Indeed, only 29.5% of respondents in MIDAS grade IV (severe disability) had regularly taken preventive medication in the past. Respondents in MIDAS grades III and IV also reported the highest numbers of hospitals visited in the past, compared with respondents in MIDAS grades I and II, which hints at an association between prolonged, inadequate management of migraine and higher levels of disability. The reported low levels of satisfaction with both preventive and acute medications among respondents could be a reflection of such un-optimal management of migraine. Thus, as reported in other studies including analyses in Asian countries [23, 39–42], there are clear unmet preventive treatment needs for Korean patients with migraine, particularly for those in most need of them. The 60+ group had the lowest prior regular use of preventive treatment. This could be explained by the development and availability of more preventive treatments in recent years, along with the natural evolution of migraine to a less disabling headache type with age [43, 44]. Lack of efficacy and undesirable side effects of preventive medication reported were in line with a previous study across six countries [45]. Similar to other findings, the proportion of respondents dissatisfied with the cost of preventive treatment was relatively low in comparison to other aspects of preventive medication use [45], and likely owing in part to the availability of generic and cheaper medications. Less than half of respondents were satisfied with the relief they had obtained within 2 h of taking acute medication in terms of being free of headache and accompanying symptoms. Such effects, along with having 24-h sustained relief, are attributes of acute treatment rated as important by patients [46–48].

We would like to note some possible limitations of the study. First, the short timeframe in which the surveys were completed may have limited the number of participants. Second, the cross-sectional nature of the study made it difficult to investigate causal relationships between variables. Third, the setting of specialized headache clinics may have introduced an element of selection bias.

## Conclusion

In conclusion, the results of our survey confirm the significant burden that Korean patients with migraine experience and the critical unmet needs with regards to diagnosis and treatment. Patient-centric intervention to reduce the diagnostic lag, increase awareness and understanding of migraine, optimize the use of medical services, enhance doctor-patient relationships and the management of migraine should be implemented to alleviate the burden of migraine.

## Supplementary Information

**Additional file 1.**

## Data Availability

Anonymized datasets generated and/or analyzed during the current study are available upon reasonable request and following the acquisition of necessary permissions.

## Abbreviations

CT: Computerized Tomography
EEG: Electroencephalogram
EF: Emotional Function
ER: Emergency Room
GI: Gastro-Intestinal
ICHD: International Classification of Headache Disorders
MIDAS: Migraine Disability Assessment
MRI: Magnetic Resonance Imaging
MSQ: Migraine-Specific Quality of Life Questionnaire
RP: Role Function-Preventive
RR: Role Function-Restrictive
SE: Standard Error
SD: Standard Deviation
TCD: Transcranial Doppler
