# Correction to: Burden of migraine and unmet needs from the patients’ perspective: a survey across 11 specialized headache clinics in Korea

**DOI:** 10.1186/s10194-021-01270-2

**Published:** 2021-06-11

**Authors:** Byung-Kun Kim, Min Kyung Chu, Soo Jin Yu, Grazia Dell’Agnello, Jeong Hee Han, Soo-Jin Cho

**Affiliations:** 1 Department of Neurology, Nowon Eulji Medical Center, Eulji University School of Medicine, Seoul, South Korea; 2 Department of Neurology, Severance Hospital, Yonsei University School of Medicine, Seoul, South Korea; 3 Lilly Korea Ltd., Seoul, South Korea; 4 Eli Lilly and Company, Sesto Fiorentino, (FI) Italy; 5 Department of Neurology, Dongtan Sacred Heart Hospital, Hallym University College of Medicine, Hwaseong, South Korea

**Correction to: The Journal of Headache and Pain 22, 45 (2021)**

**https://doi.org/10.1186/s10194-021-01250-6**

Following the publication of the original article [1], we were notified of two errors, in the legend for Fig. 3 and in the author details.

1. Fig. 3 legend: ‘Results are presented as mean percentage of respondents.’ should read ‘Results are presented as percentage of respondents.’ (the word ‘mean’ needs to be removed)

2. Author details: affiliation 4 should be ‘Eli Lilly and Company, Sesto Fiorentino (FI), Italy’ instead of ‘Eli Lilly and Company, Indianapolis, IN, USA’.

The original article has been corrected.
