# Supplementary material for: Burden of migraine and unmet needs from the patients’ perspective: a survey across 11 specialized headache clinics in Korea
Source: J Headache Pain. 2021 May 24;22(1):45. doi: 10.1186/s10194-021-01250-6 (PMC8146656; doi:10.1186/s10194-021-01250-6)
Supplement: Supplementary file 1 — Additional file 1. [file 10194_2021_1250_MOESM1_ESM.docx]

***Respondents’ narrative descriptions of migraine pain (translated from Korean)***

*Description of pain (142 respondents, 69%)*

“It is just like a woodpecker is pecking at my head, mashing my head with a hammer, and I feel like my eyes are popping out”

“Someone’s picking or cutting my head. My eyes are blown up that I feel, it’s painful for both sides of my eyes”

“The pain is like drilling my head by nail”

“I feel like there is a bomb in my head, and it is about to explode”

“Only feels like someone’s killing me hacking away at my head”

*Description of psychological difficulty, anger / discouragement / frustration (38 respondents, 18%)*

“I rather cut my head in half”

“I hope I pull out my head”

“I want to cut my head in half and throw it in cold water”

“I want to dissect my head and look in it”

“I just don’t want to live anymore, rather give up my life and die”

“I want to cut my eyes out (because of the light) and want to cut my vein because I can hear the sound of my blood passing through the back of my neck. At its worse, I just want to remove my head”

*Description of difficulty with accompanied symptoms (17 respondents, 8%)*

“I am out of my strength because of vomit or dizziness”

“Everything turns into white, because my eyes are dazzled”

“I am losing my will to live with serious dizziness”

“My head is spinning and I cannot even open my eyes”

*Description of daily disorder and sensitivity (15 respondents, 7%)*

“I cannot keep staying in my life”

“All of things in the world are just tiresome and distasteful”

“It is such sensitive and serious pain that I cannot even touch a strand of my hair”

“Feels bad and cannot sleep in bed”

*Descriptions of other (14 respondents, 7%)*

“Feel like I am tortured”

“I lost my words to be spoken”

“Must be harder pain than if you cut off my legs”

“Worse that birth pains”
